# Response to combined ipilimumab and nivolumab after development of a nephrotic syndrome related to PD-1 monotherapy

**DOI:** 10.1186/s40425-019-0655-4

**Published:** 2019-07-12

**Authors:** Valerie Glutsch, Franziska Grän, Judith Weber, Anja Gesierich, Matthias Goebeler, Bastian Schilling

**Affiliations:** Department of Dermatology, Venereology and Allergology, University Hospital Würzburg, Josef-Schneider-Str, 2, 97080 Würzburg, Germany

**Keywords:** PD-1, Immune-related adverse event, Minimal change disease, Ipilimumab, Nivolumab

## Abstract

**Background:**

High response rates of metastatic melanoma have been reported upon immune checkpoint inhibition by PD-1 blockade alone or in combination with CTLA-4 inhibitors. However, the majority of patients with a primary resistance to anti-PD-1 monotherapy is also refractory to a subsequent combined checkpoint inhibition. In BRAF wildtype patients with a primary resistance to PD-1 inhibitors, therapeutic options are therefore limited and immune-related adverse events (irAE) have to be taken into consideration when discussing a subsequent immunotherapy.

**Case presentation:**

We report the case of a 68-year-old male patient with metastatic melanoma who experienced an acute renal failure with nephrotic syndrome due to a minimal change disease developing after a single dose of the anti-PD-1 antibody pembrolizumab. A kidney biopsy revealed a podocytopathy without signs of interstitial nephritis. Renal function recovered to almost normal creatinine and total urine protein levels upon treatment with oral steroids and diuretics. Unfortunately, a disease progression (PD, RECIST 1.1) was observed in a CT scan after resolution of the irAE. In a grand round, re-exposure to a PD-1-containing regime was recommended. Consensually, a combined immunotherapy with ipilimumab and nivolumab was initiated. Nephrotoxicity was tolerable during combined immunotherapy and a CT scan of chest and abdomen showed a deep partial remission (RECIST 1.1) after three doses of ipilimumab (3 mg/kg) and nivolumab (1 mg/kg).

**Conclusion:**

This case illustrates that a fulminant response to combined checkpoint inhibition is possible after progression after anti-PD-1 monotherapy and a severe irAE.

## Background

In prospective clinical trials response rates of up to ~ 40% to anti-PD-1 monotherapy and ~ 60% for combined checkpoint inhibition (ipilimumab plus nivolumab) have been reported in patients with advanced or metastatic melanoma [1]. Unfortunately, treatment options for BRAF wildtype patients resistant to anti-PD-1 monotherapy are limited. The majority of such patients are also refractory to subsequent combined checkpoint inhibition [2, 3]. In addition, severe immune-related adverse events (irAE) related to monotherapy and possible irAE during subsequent immunotherapy must be taken into consideration when counselling these patients. Here, we report a case with a rare and severe renal irAE due to pembrolizumab monotherapy and a deep response to subsequent, well-tolerated ipilimumab and nivolumab.

## Case presentation

**Fig. 1:**
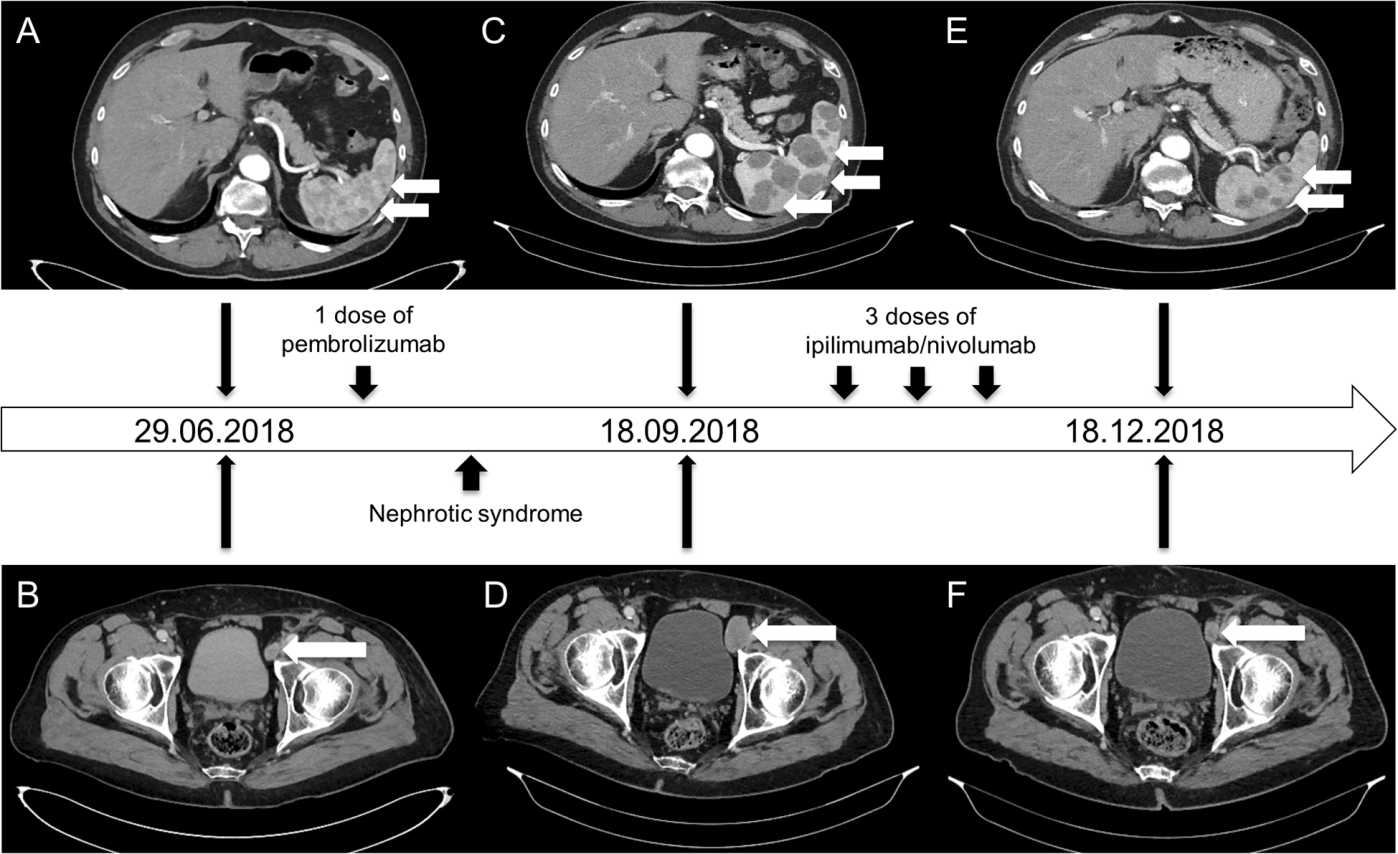
Timeline: **a-b** CT scans of the abdomen with splenic metastases and a iliac lymph node metastasis before the first dose of pembrolizumab. **c-d** CT scans of the abdomen with splenic metastases and a iliac lymph node metastasis after one dose of pembrolizumab and acute kidney injury. **e-f** CT scans of the abdomen with a fulminant response of the splenic metastases and the iliac lymph node metastasis after three doses of ipilimumab/nivolumab. White arrows indicate metastases

**Fig. 2:**
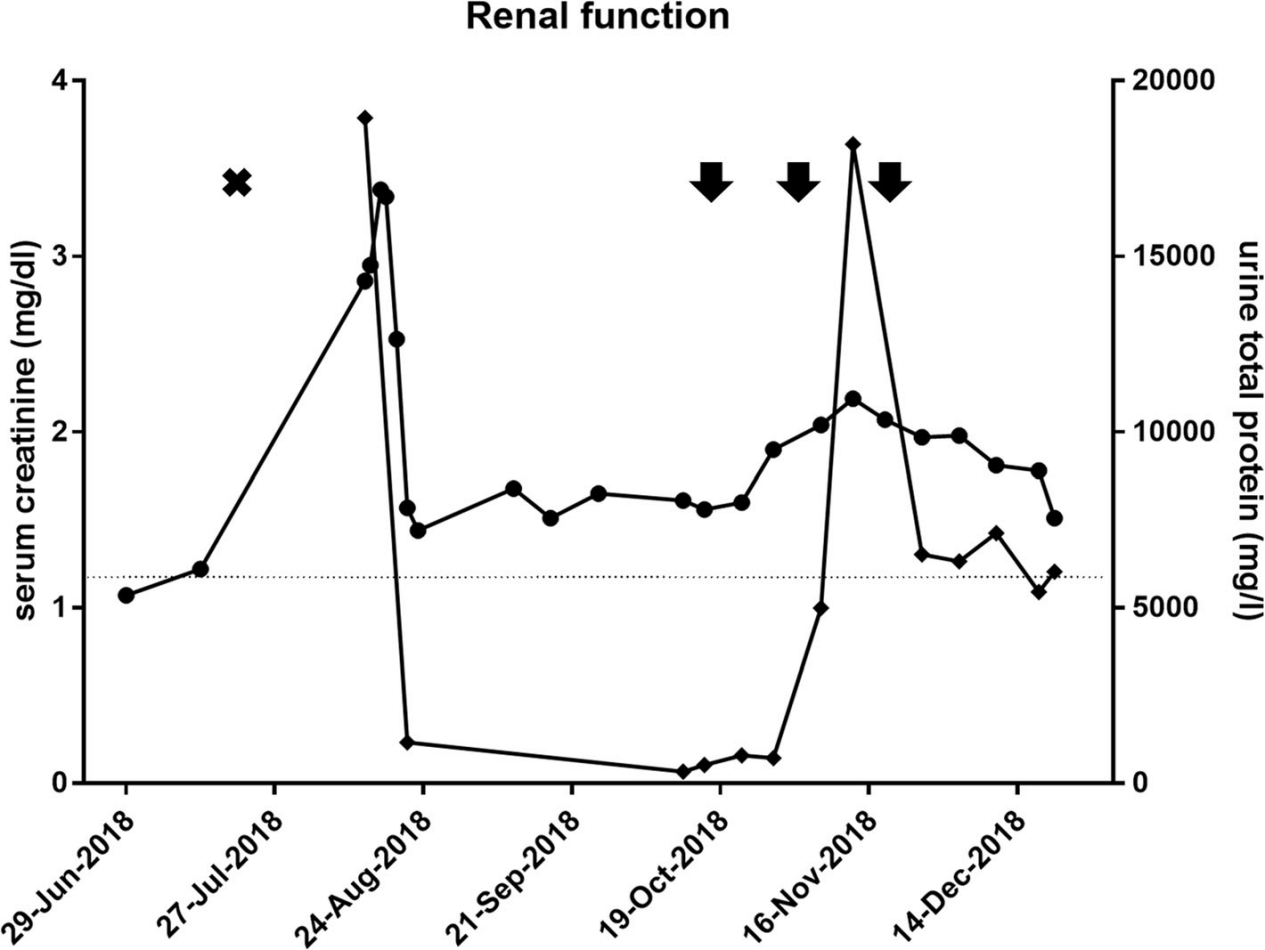
Renal function tests: Serum creatinine and urine total protein throughout pembrolizumab and ipilimumab plus nivolumab therapy. Circles show serum creatinine while diamonds represent urine total protein at given time points. Upper limits of normal (ULN): Serum creatinine (ULN = 1.17 mg/l, indicated by dashed horizontal line) and urine total protein (ULN < = 120 mg/l). Black arrows indicate applications of ipilimumab/nivolumab, black cross indicates application of pembrolizumab

A 68-year old male was diagnosed with stage IV melanoma (cM1c (0) AJCC 2017, *BRAF* wild type) with iliac lymph node, adrenal and splenic metastases (Fig. 1). Anti-PD-1 monotherapy with pembrolizumab was initiated (2 mg/kg q3w) as first-line therapy. Eighteen days after the first application of pembrolizumab, the patient reported a weight gain of 10 kg within 7 days and massive peripheral edema. Laboratory tests revealed an acute renal failure with nephrotic syndrome (creatinine 2.86 [0–1.17] mg/dl, urea 78.9 [10–50] mg/dl, potassium 5.2 [3.5–5] mmol/l, calcium 1.7 [2–2.7] mmol/l, cholesterol 399 [130–220] mg/dl, total protein 4.2 [6.6–8.7] g/dl, albumin 1.6 [3.5–5.5] g/dl). Prior to pembrolizumab, renal function tests were normal and proteinuria was absent. The patient was hospitalized and a kidney biopsy was performed. Light microscopy showed a tubular damage (presumably due to a preexistent hypertensive nephropathy) without signs for interstitial nephritis. Amyloidosis, the presence of immune complexes or complement-mediated glomerulonephritis were ruled out by immunohistochemistry. Ultimately, electron microscopy showed findings consistent with a minimal change disease. Based on these findings, an acute renal failure with nephrotic syndrome due to a minimal change disease related to pembrolizumab was diagnosed. Other risk factors for a minimal change disease (e.g. non-steroidal anti-inflammatory drugs) were not evident. Treatment with oral corticosteroids (100 mg prednisolone qd) and diuretics was initiated. Renal function recovered to creatinine levels around 1.5 mg/dl and proteinuria decreased to 329 mg/l (Fig. 2). Prednisolone was tapered over approximately 6 weeks, diuretic treatment with torasemid was reduced to a maintenance dose of 25 mg qd.

During irAE treatment, S100 serum levels increased significantly and a computed tomography (CT) scan of chest and abdomen 2 months after the single dose of pembrolizumab showed diesease progression (PD, RECIST 1.1) (Fig. 1). A grand round recommended re-exposure to a PD-1-based immunotherapy due to lacking effective therapy alternatives. The recommendation was discussed with the patient including the risk of an immunotherapy-related terminal dialysis-dependent renal insufficiency. Finally, a combined checkpoint inhibition with ipilimumab (3 mg/kg) and nivolumab (1 mg/kg) was initiated. Proteinuria and blood pressure were monitored weekly. After two applications of the combined immunotherapy, creatinine levels increased to values ~ 2 mg/dl and the patient once again showed massive proteinuria (total protein 18,200 mg/l) (Fig. 2). Fortunately, there were no signs of peripheral edema and his body weight remained stable. To curtail proteinuria, oral treatment with the ACE inhibitor ramipril was escalated to 5 mg qd.

**Fig. 3:**
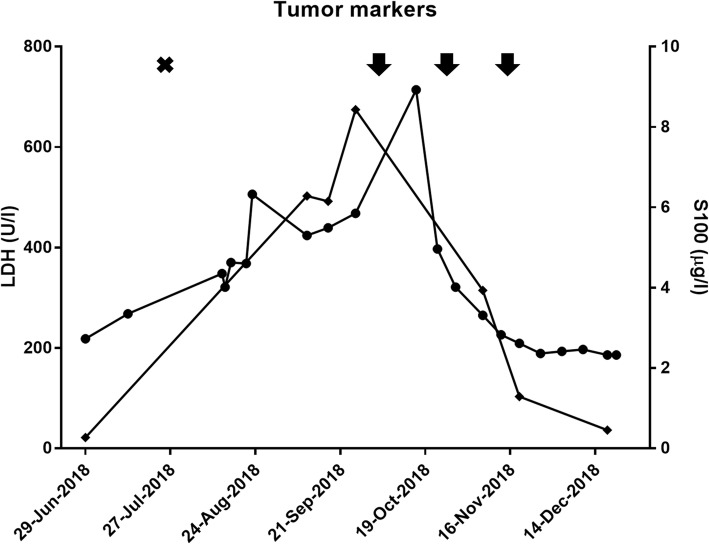
Tumor markers: Course of lactate dehydrogenase (LDH) and S100B throughout pembrolizumab and ipilimumab plus nivolumab therapy. Diamonds show serum S-100 while circles indicate LDH levels at given time points. Upper limits of normal (ULN): LDH (ULN = 250 U/l) and S100 (ULN = 0.14 μg/l). Black arrows indicate applications of ipilimumab/nivolumab, black cross indicates application of pembrolizumab

Ipilimumab and nivolumab were continued without a dose delay. Creatinine serum levels and proteinuria stabilized (Fig. 2). Nevertheless, we abstained from the fourth dose after another nephrological consultation and due to sonographic and serological signs for response becoming evident. An ultrasound of the abdomen performed after two doses of ipilimumab and nivolumab had already shown a shrinkage of the iliac lymph node metastasis and S100 serum levels were dropping (Fig. 3). A CT scan after three doses of combined checkpoint inhibition confirmed a deep partial response (PR, RECIST 1.1) with regression of all known visceral and lymph node metastases. There were no signs for new thoraco-abdominal or brain metastases (MRI). Due to the renal irAE during anti-PD-1 monotherapy and a deep PR after three doses of ipilimumab and nivolumab, we refrained from a maintenance treatment with nivolumab.

## Discussions and conclusions

The frequency of renal adverse events related to anti-PD-1 therapy is very low [4–6]. Interstitial nephritis with predominant tubulointerstitial injury is the most common presentation of an acute kidney injury related to anti-PD-1 therapy [4, 7, 8], whereas an acute renal failure with nephrotic syndrome due to a minimal change disease is rare. So far, only two cases of nephrotic syndrome with minimal change disease secondary to therapy with an anti-PD-1 antibody have been reported [9, 10]. Both patients received pembrolizumab for Hodgkin’s lymphoma (HL). In contrast to HL, malignant melanoma is not known to induce minimal change disease itself [11–13]. Thus, the acute kidney injury in our patient was related to pembrolizumab most likely. Consistent with the two cases reported and guidelines for managing irAE, immunotherapy was stopped and both creatinine as well as proteinuria improved after administering systemic glucocorticoids. In case of an immune-related acute kidney injury grade 3 according to the Common Toxicity Criteria of Adverse Events (CTCAE) treatment with methylprednisolone 0,5–1 mg/kg daily is recommended and creatinine levels should be monitored every 2 to 3 days [5]. In case of unclear clinical findings a kidney biopsy and a nephrology consultation are warranted [5].

Most melanoma patients resistant to nivolumab or pembrolizumab monotherapy are also refractory to a subsequent combined immunotherapy with ipilimumab plus nivolumab [2, 3]. However, there are case reports of fulminant responses to combined checkpoint inhibition after failure of anti-PD-1 monotherapy despite unfavorable predictive factors such as elevated lactate dehydrogenase (LDH) [14]. Besides, there are reports that immunotherapy is safe in patients with an impaired renal function due to other underlying diseases [15]. In a process of participatory decision-making considering possible risks (e.g. dialysis-dependent renal failure) and alternative treatment options (PD-1 monotherapy with nivolumab, CTLA-4 monotherapy with ipilimumab or chemotherapy with dacarbazine), combined checkpoint inhibition with ipilimumab and nivolumab was initiated and led to a deep response without new toxicites.

This unique case demonstrates that a response to combined checkpoint inhibition is possible after disease progression after anti-PD-1 monotherapy and that application of an anti-PD-1-based treatment after a severe irAE during anti-PD-1 monotherapy might be worthwhile. Keeping in mind that a response to ipilimumab plus nivolumab is still rare after disease progression after anti-PD-1 monotherapy [2, 3], this treatment sequence should only be chosen in case of lacking effective treatment alternatives such as a targetable driver mutation.

## Data Availability

Not Applicable.
